# Null Function of *Npr1* Disturbs Immune Response in Colonic Inflammation During Early Postnatal Stage

**DOI:** 10.1007/s10753-022-01702-4

**Published:** 2022-07-07

**Authors:** Changkun Long, Hongfei Liu, Wenxing Zhan, Liping Chen, Andong Wu, Lin Yang, Shenghan Chen

**Affiliations:** 1 Vascular Function Laboratory, Human Aging Research Institute and School of Life Science, and Jiangxi Key Laboratory of Human Aging, Nanchang University, Nanchang, 330031 China; 2 Aging and Vascular DiseasesSchool of Life Scienceand Jiangxi Key Laboratory of Human Aging, Human Aging Research Institute, Nanchang University, Nanchang, 330031 China; 3 Department of Nephrology, Jiangxi Provincial People’s Hospital, Affiliated to Nanchang University, Nanchang, 330006 China

**Keywords:** CD4^+^ T cells, colitis, colonic inflammation, immune response, *Npr1*

## Abstract

Natriuretic peptide receptor 1 (NPR1) is conventionally known as a regulator of vascular homeostasis. Here, we generated an *Npr1* knockout mouse model with CRISPR/Cas9 technology and found that homozygous mice (*Npr1*^−/−^) exhibited weight loss and poor survival rate during early postnatal stage. Careful examination revealed unexpectedly that *Npr1*^−/−^ mice developed colitis characterized by shortened colon, evident colonic mucosal damage, increased histopathological score, and higher colonic expression of proinflammatory cytokines interleukin-1B (IL1B) and -6 (IL6). RNA-sequencing analysis revealed that differentially expressed genes were prominently enriched in the biological pathways related to immune response in both spleen and colon of *Npr1*^−/−^ mice. Cytofluorimetric analysis demonstrated that leukocytes in the spleen were significantly increased, particularly, the populations of neutrophil and CD3^+^ T cell were elevated but CD4^+^ T cells were decreased in *Npr1*^−/−^ mice. Administration of 8-Br-cGMP, a downstream activator of NPR1, restored these immune-cell populations disturbed in *Npr1*^−/−^ mice and lessened the colitis-related phenotypes. To validate the involvement of *Npr1* in colitis, we examined another mouse model induced by dextran sodium sulfate (DSS) and found a decreased *Npr1* expression and shifted immune-cell populations as well. Importantly, 8-Br-cGMP treatment exhibited a similar effect in the restoration of immune-cell populations and attenuation of colonic inflammation in DSS mice. Our data indicate that loss of *Npr1* possibly interrupts immune response, which is critical to the pathogenesis of colitis in the early life.

## INTRODUCTION

The dysfunctional postnatal immune response may lead to rising morbidity and mortality in the early life [1]. Inflammatory bowel disease (IBD) is a gut disorder featured by chronic inflammation and considered an immune-mediated disease [2]. IBD has an increasing incidence in children globally and early-onset disease is more severe in infants and young children [3–7]. Studies show that pediatric colitis present failure to thrive and increased percentage of early death [8, 9]. Also, pediatric-onset IBD increases the risk of thromboembolism, colon cancer, and depressive disorder, which is responsible for higher mortality during adulthood [4, 10–12], but the underlying mechanisms are complicated.

Natriuretic peptide receptor 1 (NPR1) (also known as NPRA or GC-A) is a membrane-bound receptor for atrial natriuretic peptide (ANP) and B-type natriuretic peptide (BNP), which is expressed in many cell types and tissues including heart, vasculature, spleen, and gut [13–15]. Upon activation by ANP or BNP, NPR1 stimulates the production of cyclic guanosine monophosphate (cGMP) that promotes vascular tone and volume homeostasis by activation of protein kinase cGMP-dependent 1 (PKG), thereby controlling blood pressure and maintaining cardiovascular homeostasis [16, 17]. Mice with homozygous deletion of *Npr1* (*Npr1*^−/−^) have shown hypertension, cardiac hypertrophy, and sudden death [18]. But how NPR1 contributes to vascular aging and related cardiovascular diseases remains not fully illustrated.

We initially generated *Npr1*^−/−^ mouse line to assess its contribution to age-related vascular function and observed very high death rate during postnatal period rather than in the adulthood as reported previously [18]. When close-up examining these mice, we unexpectedly found the morphological changes in the spleen and colon. Thus, we decided to test the hypothesis that *Npr1* deficiency impairs the immune system that further contributes to colitis in different models.

## MATERIALS AND METHODS

### Mouse Models

*Npr1*^−/−^ mice were generated in the C57BL/6 background using CRISPR/Cas9 technique (Bioray Laboratories Inc, China). The genotypes were validated by PCR-based strategies. Mice at the age of 4 weeks were used for spleen and colon tissue collection. DSS-induced colitis mice were fed with 6% dextran sodium sulfate (DSS) (Bmassay, China) in water every other day for 7 days. Simultaneously, the control mice were fed with water only. The study was conducted only with the male mice. All procedures in this study were conducted according to the Guide for the Care and Use of Laboratory Animals from the Human Aging Research Institute of Nanchang University.

### RNA Sequencing and Data Analysis

Total RNA was isolated from mouse spleen and colon tissues using Trizol reagent (Invitrogen, USA). mRNA was purified by Oligo(dT)-attached magnetic beads and consequently prepared into single-strand circle DNA as the final library. After amplification, DNA nanoballs were made and then loaded onto the patterned nanoarray for sequencing using BGIseq500 platform (BGI-Shenzhen, China). The sequencing results were filtered and the clean reads were mapped to the Mus musculus genome. Afterward, the data were analyzed by Gene Ontology (GO) enrichment analysis. A heatmap was created using Package “pheatmap” (v1.0.8) based on the gene expression value.

### Fluorescence-Activated Cell Sorting (FACS) Analysis

The cells from spleen were harvested from WT and *Npr1*^−/−^ mice and washed with cold PBS buffer. Then, the cells were made a suspension of 1 × 10^6^ cells per milliliter in PBS and divided into two groups. One group of cells were incubated with mouse anti-CD45, anti-CD3, anti-CD8, anti-CD11B, anti-Ly-6G, and anti-NK1.1 (BD Biosciences, USA) at 4 °C for 15 min. The other group of cells were first incubated with mouse anti-CD3, anti-CD4, and anti-CD8 at 4 °C for 15 min and then blocked with 1 × TF Fix/Perm working solution for 40 min. Subsequently, the cells were incubated with mouse anti-FOXP3 (BD Biosciences, USA). Finally, the cells from both groups were washed with PBS and inspected using FACSVerse flow cytometer (BD Biosciences, USA). The scatter plots were created and analyzed by FlowJo v10.0.

### 8-Br-cGMP Treatment

For administration of 8-Br-cGMP (MedChemExpress, China), two groups of *Npr1*^−/−^ mice (*n* = 4 per group) at the age of 8 weeks were used. The experimental group received 0.1 mmol/L of 8-Br-cGMP in saline by tail-vein injection each week for 4 consecutive weeks. The control group received saline with the same procedure. For DSS-induced colitis mouse model with administration of 8-Br-cGMP, WT mice (8 weeks) were divided into three groups (*n* = 10 per group) including two experimental groups (DSS + saline and DSS + 8-Br-cGMP) and one control group. Prior to the induction of colitis, mice from each group were injected with one dose of 0.1 mmol/L of 8-Br-cGMP in saline or saline only through tail vein, respectively.

### Blood Pressure Monitor

The measurement of systolic blood pressure was performed in mice by non-invasive tail-cuff device IITC (Life Science, USA). Prior to recorded measurement, mice underwent adaptation training for 1 week. Afterward, the blood pressure was measured with the inflation and deflation reading at an interval of 15 s, and 10 consecutive readings were recorded. The monitor was carried out for 3 consecutive days for each mouse.

### RNA Isolation and RT-PCR Analysis

The spleen and colon tissues from WT and *Npr1*^−/−^ mice were isolated and homogenized in TRIzol reagent (Bmassay, China) for extracting RNA. cDNA was obtained from 500 μg of total RNA by reverse transcriptase kit (Zomanbio, China). Levels of mRNA expression of Il6, Gapdh were analyzed by RT-PCR. The primer sequences were as follows:

- mouse-β-actin-F: 5ʹ-GCCGACAGGATGCAGAAGGAGATCA-3ʹ.
- mouse-β-actin-R: 5ʹ-AAGCATTTGCGGTGGACGATGGA-3ʹ.
- mouse-Il6-F: 5ʹ-GTCAGGGGTGGTTATTGC-3ʹ.
- mouse-Il6-R: 5ʹ-TCATCACTGGTCTTTTGGAG-3ʹ.

### Enzyme-Linked Immunosorbent Assay

Serum was collected from WT and *Npr1*^−/−^ mice at the age of 4 weeks. IL1B and IL6 were measured by duoset enzyme-linked immunosorbent assay kits (Proteintech, China) according to the manufacturer manual.

### Histological Analysis

The ascending colon, the most commonly affected tissues [19], from mice was fixed with 4% paraformaldehyde for 24 h, and then embedded in paraffin. The sample sections were prepared in 5 μm thickness and later analyzed by H&E staining.

### Immunofluorescent Staining

Tissue fixation was carried out in 4% paraformaldehyde solution for 15 min, and then permeabilization in PBS with 0.25% Triton X-100 for 10 min. After blocking, the samples were incubated with primary antibodies NPR1 (1:100, Thermo, USA), CD3 (1:200, Proteintech, China), CD4 (1:100, Abcam, USA), CD8 (1:500, Abcam, USA) at 4 °C overnight, and followed by Cyanine3 Donkey anti-rabbit IgG (1:200, Biolegend, USA) for 1 h in the dark at room temperature. Hoechst 33,342 (1:100, Beyotime, China) was used for nuclei staining. Images were taken with Zeiss confocal microscope (LSM800, Zeiss, Germany).

## RESULTS

### *Npr1*^−/−^ Mice Exhibit Weight Loss, Elevated Blood Pressure, and Postnatal Death

**Fig. 1:**
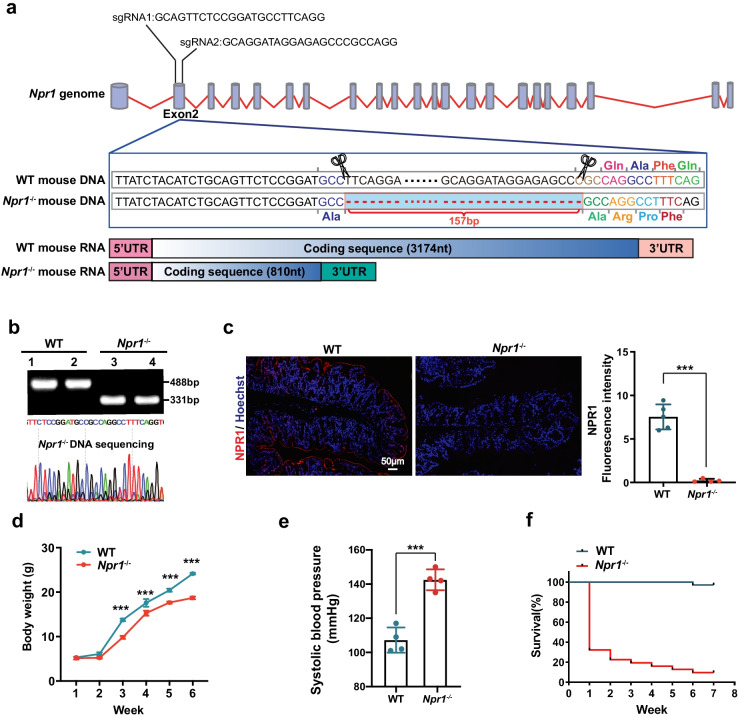
Characteristics of *Npr1*^−/−^ mice. **a** Schematic diagram of the construction of *Npr1*^−/−^ mice. **b** PCR analysis for mouse genotyping and DNA sequencing. DNA bands at 488 and 331 bp represent normal and targeted *Npr1* allele, respectively. **c** Immunofluorescent staining for NPR1 expression (red) and nuclei (blue) in the colon tissue sections from WT and *Npr1*^−/−^ mice (4 weeks, *n* = 4–5). Quantitative data obtained by mean fluorescence intensity of each sample. **d** Body weight curves in WT and *Npr1*^−/−^ mice (*n* = 7). **e** Blood pressure in WT and *Npr1*^−/−^ mice (*n* = 4). **f** Survival curves of WT and *Npr1*^−/−^ mice (*n* = 31–35). Values are mean ± S.D. *** *p* < 0.001.

*Npr1* knockout mice were generated with a deletion of 157 bp in exon 2 of *Npr1* gene creating a frameshift onto C57BL/6 background using CRISPR/Cas9 technique (Fig. 1a). The homozygous deletion of *Npr1* in mice was verified by PCR genotyping and DNA sequencing (Fig. 1b), as well as immunofluorescent staining (Fig. 1c). Upon generating this mouse line, we observed that the body weight was reduced with increased age in *Npr1*^−/−^ mice compared to wild-type (WT) mice after birth (Fig. 1d). As expected, *Npr1*^−/−^ mice showed higher blood pressure (Fig. 1e). Strikingly, the survival rate of *Npr1*^−/−^ mice was 35% at 1 week after birth and declined to 15% at the age of 4 weeks, reaching the lowest level at 7th week (Fig. 1f). There was no change on litter size in *Npr1*^−/−^ mice. These results indicated that loss of *Npr1* promoted growth retardation, hypertension, and early postnatal death in mice.

### *Npr1*^−/−^ Mice Show Very Early Onset Colitis that Is Alleviated by 8-Br-cGMP

**Fig. 2:**
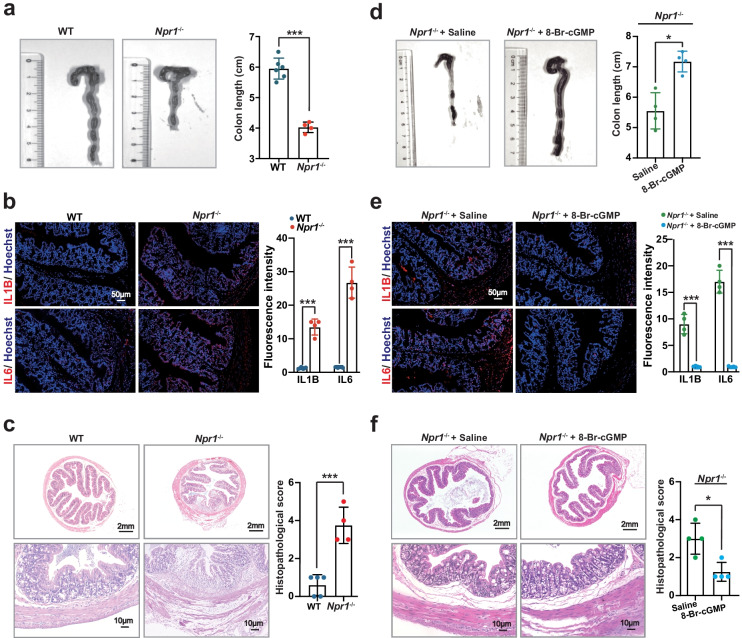
Early onset colitis in *Npr1*^−/−^ mice at the age of 4 weeks. **a** The colon length from WT and *Npr1*^−/−^ mice (*n* = 4–6). **b** IL1B and IL6 expressions in the colon from WT and *Npr1*^−/−^ mice (*n* = 4). **c** Histopathological changes in the colon tissue sections from WT and *Npr1*^−/−^ mice (*n* = 4–5). **d** The colon length from *Npr1*^−/−^ mice treated with 8-Br-cGMP or saline as a control (*n* = 4). **e** IL1B and IL6 expression in the colon from *Npr1*^−/−^ mice treated with 8-Br-cGMP or saline (*n* = 4). **f** Histopathological changes in the colon tissue sections from from *Npr1*^−/−^ mice treated with 8-Br-cGMP or saline (*n* = 4). Values are mean ± S.D. * *p* < 0.05; *** *p* < 0.001.

Next, we examined whether *Npr1* contributes to the development of colitis in mice at the age of 4 weeks. We found the shortened colon (Fig. 2a) and increased IL1B and IL6 level in the colon tissue (Fig. 2b) in *Npr1*^−/−^ mice. At the histological level, the colonic lesion was observed and the histopathological score was significantly higher in *Npr1*^−/−^ mice than that in WT (Fig. 2c). However, by administration of 8-Br-cGMP (cGMP analog), the colon length and IL1B and IL6 expressions were recovered (Fig. 2d and e) and the mucosal damage was attenuated (Fig. 2f). These data suggested that absence of *Npr1* caused colitis in the early postnatal period in mice.

### Immune-Related Differentially Expressed Genes (DEGs) Are Identified in *Npr1*^−/−^ Mice

**Fig. 3:**
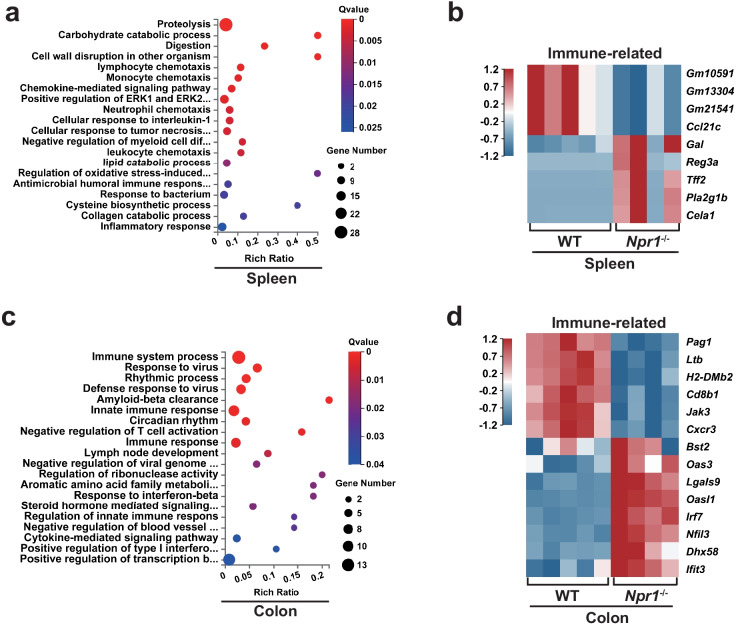
Identification of DEGs in the spleen and colon from *Npr1*^−/−^ mice at the age of 4 weeks. **a** GO enrichment analysis of DEGs (log2 fold-change > 1.5 and *p* < 0.05) in the spleen from WT and *Npr1*^−/−^ mice (*n* = 3). The rich ratio is the ratio of the DEG number and the number of all genes in a certain enrichment pathway. The dot size denotes the number of DEGs, the colors denotes the adjusted Q-value range. **b** A heat map for immune-related DEGs in the spleen from WT and *Npr1*^−/−^ mice (*n* = 3). The color intensity indicates the relative expression levels of up-regulated (red) and down-regulated (blue) DEGs. **c** GO enrichment analysis of DEGs in the colon from WT and *Npr1*^−/−^ mice (*n* = 3). **d** A heat map for immune-related DEGs in the colon of WT and *Npr1*^−/−^ mice (*n* = 3).

To investigate whether *Npr1* deficiency is impact on the immune response in the early postnatal stage, we carried out RNA sequencing for the spleen and colon from WT and *Npr1*^−/−^ mice at the age of 4 weeks. The data was assessed by GO enrichment analysis. We found that DEGs in the spleen were enriched in biological processes including lymphocyte chemotaxis, monocyte chemotaxis, neutrophil chemotaxis, and chemokine-mediated signaling pathway (Fig. 3a). A total of 9 DEGs were clustered in these pathways, in which 5 genes including *Pla2g1b* (phospholipase A2 group 1B) were up-regulated and 4 genes were down-regulated (Fig. 3b). Likewise, DEGs in the colon were involved in the signaling pathways related to immune system process, innate immune response, negative regulation of T-cell activation, and immune response (Fig. 3c). There were 14 DEGs identified from these pathways, in which 8 genes including *Lgals9* (galectin 9) were up-regulated and 6 genes such as *Jak3* (Janus kinase 3) were down-regulated (Fig. 3d). These data confirmed that lack of *Npr1* altered the expression of genes related to immune regulation in the spleen and colon during early life of mice.

### *Npr1*^−/−^ Mice Display the Composition Changes of Immune Cells in the Spleen

**Fig. 4:**
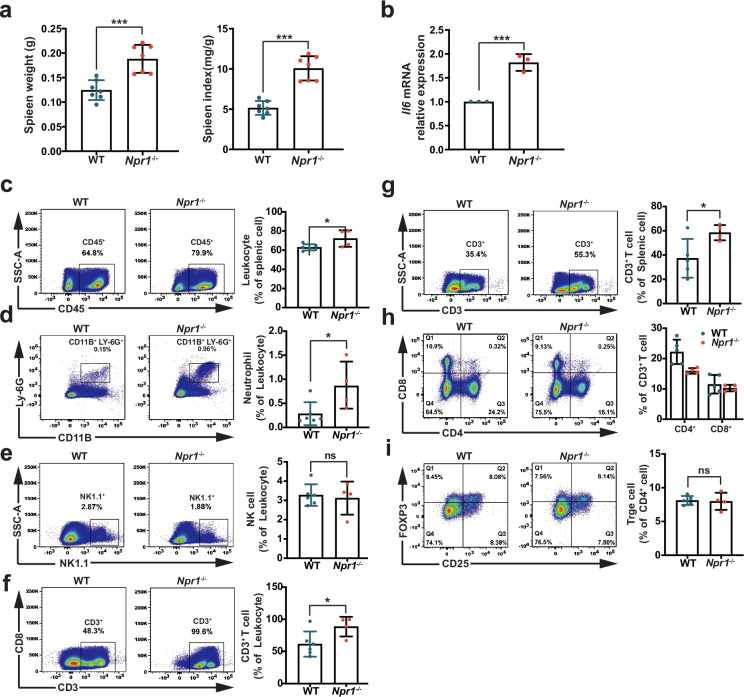
Changes of immune cell composition in the spleen from *Npr1*^−/−^ mice at the age of 4 weeks. **a** The spleen weight and index from WT and *Npr1*^−/−^ mice (*n* = 7). Spleen index was generated as spleen weight (mg)/body weight (g). **b** Expression of *Il6* mRNA in the spleen from WT and *Npr1*^−/−^ mice (*n* = 3). **c** Leukocytes defined as CD45^+^ in the splenic immune cells from WT and *Npr1*^−/−^ mice (*n* = 4–6). Population of neutrophils featured by **d** CD11B^+^LY-6G^+^, **e** NK cells, and **f** T cells featured by CD3^+^ from leukocytes in the spleen from WT and *Npr1*^−/−^ mice (*n* = 4–6). **g** Population of CD3^+^ T cells in the splenic cells from WT and *Npr1*^−/−^ mice (*n* = 4–5). **h** Population of CD4^+^ T and CD8^+^ T differentiated from CD3^+^ T cells from WT and *Npr*1^−/−^ mice. **i** Population of Treg cells from CD4^+^ T cells in WT and *Npr1*^−/−^ mice. Values are mean ± S.D. * *p* < 0.05; *** *p* < 0.001; ns, nonsignificant.

To further understand the immune response in *Npr1*^−/−^ mice, we assessed the composition of immune cells in the spleen of mice at the age of 4 weeks. We found enlarged spleen, increased spleen weight and elevated spleen index (Fig. 4a), and up-regulated splenic proinflammatory cytokine Il6 (Fig. 4b) in *Npr1*^−/−^ mice. Fluorescence-activated cell sorting (FACS) analysis showed that leukocytes were significantly increased in the spleen of *Npr1*^−/−^ mice (Fig. 4c), suggesting that leukocyte infiltration may cause the splenomegaly. Among leukocytes, increased proportion was found in neutrophils (Fig. 4d), but not in natural killer (NK) cells (Fig. 4e). Moreover, the elevated proportion of CD3^+^ T cells was observed among the leukocytes (Fig. 4f) as well as splenic cells (Fig. 4g) in *Npr1*^−/−^ mice, in which the population of CD4^+^ T cells and CD8^+^ T cells differentiated from CD3^+^ T cells was further analyzed. The results showed that the proportion of CD4^+^ cells, but not CD8^+^ cells, was lower in *Npr1*^−/−^ mice than that in WT mice (Fig. 4h). Additionally, the population of Treg cells from CD4^+^ T cells showed no difference in *Npr1*^−/−^ and WT mice (Fig. 4i). All the results indicated that *Npr1* deficiency impaired the immune response and differentiation of immune cells in the early development of mice.

### T-Cell Population Is Disturbed but Restored by 8-Br-cGMP in the Colon from *Npr1*^−/−^ Mice

**Fig. 5:**
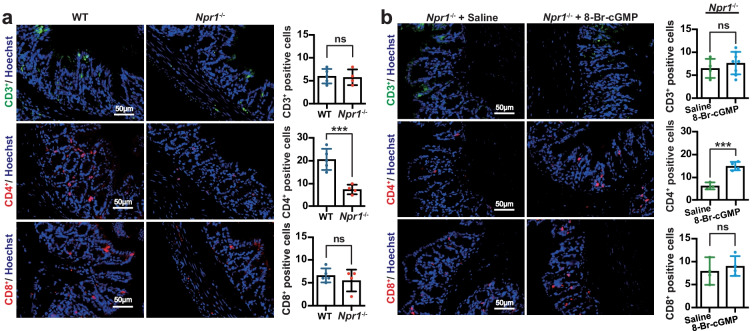
Restructuring of T-cell subpopulation in the colon tissue from *Npr1*^−/−^ mice. Immunofluorescent staining for CD3^+^, CD4^+^, and CD8^+^ in **a** WT and *Npr1*^−/−^ mice (4 weeks, *n* = 4–5) and **b** *Npr1*^−/−^ mice treated with 8-Br-cGMP or saline as a control (12 weeks, *n* = 4). Values are mean ± S.D. * *p* < 0.05; ** *p* < 0.01; *** *p* < 0.001; ns, nonsignificant.

To test whether T-cell subpopulations are changed in *Npr1*^−/−^ mice, we carried out immunofluorescent staining in the colon tissue sections. We observed that CD4^+^ T cells were significantly decreased in *Npr1*^−/−^ mice at the age of 4 weeks, but CD3^+^ and CD8^+^ T cells were not altered (Fig. 5a). A decreased CD4^+^ T-cell population was restored after administration of 8-Br-cGMP (Fig. 5b), suggesting that *Npr1* deficiency shifted T-cell population in the process of colitis.

### NPR1 Expression Is Reduced in the Mouse Model of Colitis Induced by DSS

**Fig. 6:**
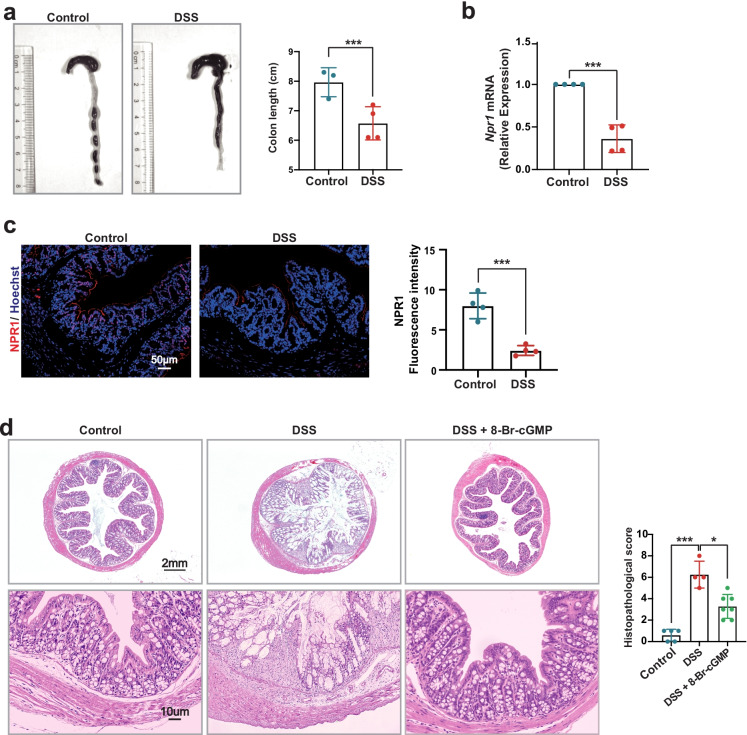
NPR1 expression in DSS-induced colitis mouse model. **a** The colon length from control and DSS-treated mice (8 weeks, *n* = 3–4). **b** *Npr1* mRNA expression in the colon from control and DSS-treated mice (8 weeks, *n* = 3–4). **c** Immunofluorescent staining for NPR1 expression (red) and nuclei (blue) in the colon tissue sections from control and DSS-treated mice (8 weeks, *n* = 3). Quantitative data obtained by mean fluorescence intensity of each sample. **d** Histopathological changes in the colon tissue sections from control, DSS-treated mice, and DSS-treated mice with 8-Br-cGMP administration (9 weeks, *n* = 4–7). Values are mean ± S.D. * *p* < 0.05; *** *p* < 0.001.

To validate NPR1 implicating in the colonic inflammation, we established DSS-induced colitis mouse model that is extensively used because of the most similarity to human ulcerative colitis [20]. These mice exhibited the shortened colon length (Fig. 6a), decreased NPR1 mRNA, and protein expression level (Fig. 6b and c). We also found colonic mucosal damage and elevated histopathological score, but these phenotypes were improved by treatment of 8-Br-cGMP (Fig. 6d). All the data indicated that NPR1 may play a vital role in the experimental colitis.

### Interrupted T-Cell Population Is Restored by 8-Br-cGMP in DSS Mice

**Fig. 7:**
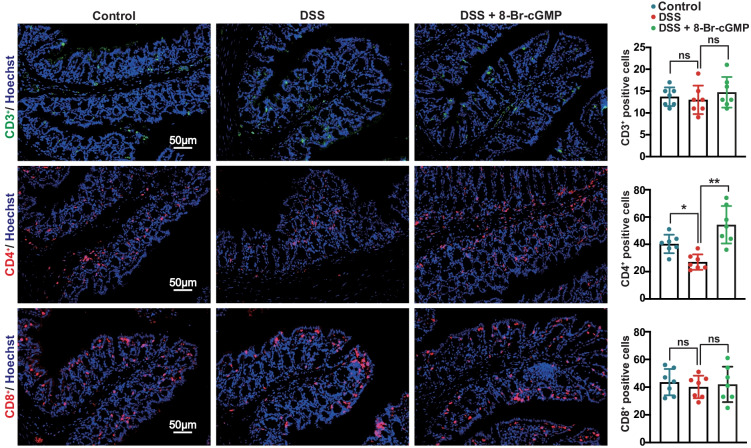
Restructuring of T-cell subpopulation in the colon tissue from DSS-treated mice. Immunofluorescent staining for CD3^+^, CD4^+^, and CD8^+^ in control, DSS-treated mice, and DSS-treated mice with 8-Br-cGMP administration (9 weeks, *n* = 7). Values are mean ± S.D. * *p* < 0.05; ** *p* < 0.01; ns, nonsignificant.

To confirm the involvement of immune cells in colitis, we examined T-cell population in the colon tissue section from DSS mouse model using immunofluorescent staining. Consistent with the results in *Npr1*^−/−^ mice, the population of CD4^+^ T cells was decreased in DSS mice compared to the control mice, but reduced CD4^+^ T-cell population was restored by 8-Br-cGMP treatment (Fig. 7, middle panel). However, CD3^+^ (Fig. 7, upper panel) and CD8^+^ T-cell population (Fig. 7, bottom panel) remained unchanged. These results suggested that *Npr1* is involved in modulation of T-cell subpopulations in the colonic inflammation.

## DISCUSSION

In this study, we found that null function of *Npr1* disrupts the immune response that further leads to colitis in mice at the early life stages.

Pediatric IBD at very early onset accounts for about 25% of the cases [21], which results in growth retardation and increased mortality in later life [10, 22]. The pathogenesis of IBD is multifaceted with the involvement of genetic effect and immune response [23]. Here, we demonstrate in both *Npr1*^−/−^ and DSS mouse models that loss of *Npr1* disturbs the immune response with shifted population of immune cells, which causes experimental colitis during postnatal period.

In our study, *Npr1*^−/−^ mouse model was initially utilized for evaluation of vascular aging. Unexpectedly, we found abnormal morphology of spleen and colon. To investigate the underlying mechanism for this phenotype, we conducted RNA sequencing for these two tissue samples. Based on the data analysis, we found a diverse set of DEGs for the spleen and colon of *Npr1*^−/−^ mice, which are linked to the signaling pathways of the immune response. Among those up-regulated DEGs, PLA2G1B has been reported to decline the survival rate of CD4^+^ T cells *in vitro* and induce dysfunction of CD4^+^ T cells but CD8^+^ T cells, which inhibited the sensitivity of CD4^+^ T cells to inflammatory cytokines such as IL2, IL4, and IL7 [24]. Another gene *LGALS9* is an important molecule involved in the immune response and development. Increased *LGALS9* expression in humans is positively correlated with augmentation of T-cell markers and proinflammatory cytokines such as IL1B and IL6 in the affected intestines, which also has positive correlation to the severity of colitis [25]. Conversely, lack of *Lgals9* in mice showed less gut inflammation reflected by colonic morphology and histology [25]. In a cluster of down-regulated DEGs, *Jak3* is the most studied gene involved in immunological function. *Jak3* deficiency exacerbates colonic inflammation with shortened colon, marked mucosal impairment, elevated neutrophil, and increased Il6 [26, 27], which is consistent to the phenotype observed in our *Npr1*^−/−^ mouse model. In mice lacking *Jak3*, ENaC (epithelial Na^+^ channel) activity is damaged and accompanied by sodium depletion in the colonic epithelium [28]. Therefore, NPR1 may interplay with these genes during colonic inflammation in the postnatal period of mice.

Spleen is the largest immune organ. It serves as a critical location for immune surveillance and response, while it is not totally developed until the age of 2 years in human [1, 29], equivalent to 2–3 weeks in mice [30]. Available evidence indicates that the postnatal life is a critical time frame for immune development and homeostasis [31]. The impairment of immune response in the early neonatal period may contribute to immune-related or inflammatory disorder [32]. We found enlarged spleen, increased spleen weight, elevated spleen index, and altered immune cell profiles in *Npr1*^−/−^ mice at postnatal stage. An elevated population of leukocytes and neutrophils is a strong evidence for splenomegaly caused by immune cell infiltration. In the early neonatal period, neutrophils undergo maturation in functionality [1]. Of note, neutrophils are the important population of the innate leukocytes, which release some cytokines such as Il1b [33]. Loss of granulocyte–macrophage colony-stimulating factor results in highly expressed Il1b and Il6 in the spleen and increased mortality in mice [34]. Il6 also mediates recruiting leukocytes to the spleen and enhances Il6 level, causing early postnatal death [35, 36]. Consistent with the results from previous reports, we observed that IL6 level was significantly increased in the spleen of *Npr1*^−/−^ mice. Similar to the spleen, increased IL1B and IL6 were found in the colon of *Npr1*^−/−^ mice. Our findings support that impaired mucosal immune response promotes local proinflammatory cytokine, causing colonic inflammation [37, 38].

It is well documented that T cells are essential for the immune response [39]. The immune response is modulated by T-cell activation pathway [23, 32]. Through cytofluorimetric analysis, we found that the populations of CD3^+^ T cells were elevated but CD4^+^ T cells were decreased in the spleen from *Npr1*^−/−^ mice. CD3^+^ T cells comprise CD4^+^ and CD8^+^ T cells that are important for assessing immunological function and pathological alterations [40]. CD3^+^ T cell is one of the subsets in the splenic leukocytes, accounting for one-fourth of all splenocytes [34, 41]. Studies show that dysfunction of CD3^+^ mediates gut inflammation with higher Il6 and Il1b levels, while anti-CD3 antibody remarkably decreases inflammatory phenotype by inhibiting the production and secretion of proinflammatory cytokine [42, 43]. Furthermore, we also found that the population of CD4^+^ T cells was reduced in the colon tissue from *Npr1*^−/−^ and DSS mice. Administration of 8-Br-cGMP, a downstream activator of NPR1, restored these immune-cell populations disturbed in *Npr1*^−/−^ mice. It has been reported that CD4^+^ T cells with large population are presented in the lamina propria of gut, which are important for immune system homeostasis and inflammation [44]. The decline of CD4^+^ cell amount and function predisposes inflammation and infection [45]. Stimulation of CD4^+^ T cells to produce IL-10 inhibits the inflammatory response in the process of colitis [46]. In IBD patients and colitis mice, the population of CD4^+^ T cells is significantly decreased [47, 48]. A previous study has shown that NPR1 is expressed in CD4^+^ T cells, which controls the development of Th17 cells by ANP stimulation [49]. These suggest that NPR1 regulates the immune response mediated by restructuring T-cell subpopulations to promote early development of colitis.

Our data from two mouse models demonstrate that *Npr1* deficiency causes a colonic inflammation phenotype accompanied with growth retardation, resembling pediatric onset colitis [8, 9, 50]. When administrating PKG activator 8-Br-cGMP, the phenotype of colitis was attenuated in *Npr1*^−/−^ and DSS mice. A very recent study has reported that decreased levels of ANP and NPR1 exacerbate ulcerative colitis in humans and the experimental mice. Mechanistically, this inflammatory response is mediated by ANP stimulator of interferon gene (STING) cascade in the process of colitis [15].

In the clinical perspective, pediatric IBD patients seem to have more serious disease course with adverse consequences including perianal fistulae, pancolitis, growth failure, psychosocial disorder, and resistant to medical treatment [4, 51, 52]. For the immune-related disease, the biologic therapy has been emerged as an improved therapeutic approach [53]. Early intervention with biologic therapies provides more effective outcomes for pediatric IBD [4, 54]. The biologic agents include monoclonal antibodies and fusion proteins, such as anti-TNF-α, anti-integrin, and anti-interleukin 12/23 [53]. Our study provides more supportive evidence for intervention strategy in IBD. In sum, our findings highlight that loss of *Npr1* possibly disturbs the immune response leading to colitis in the early life. Importantly, NPR1 may become a potential therapeutic target for anti-colonic inflammation beyond regulating vascular homeostasis such as blood pressure.

## Data Availability

All the data of this study are included in the manuscript.
